# Identification of Early Hippocampal Dynamics during Recognition Memory with Independent Component Analysis

**DOI:** 10.1523/ENEURO.0183-23.2023

**Published:** 2024-03-29

**Authors:** Víctor J. López-Madrona, Agnès Trébuchon, Ioana Mindruta, Emmanuel J. Barbeau, Andrei Barborica, Costi Pistol, Irina Oane, F. Xavier Alario, Christian G. Bénar

**Affiliations:** ^1^Inst Neurosci Syst, INS, INSERM, Aix Marseille Univ, Marseille 13005, France; ^2^Epileptology and Cerebral Rhythmology, APHM, Timone Hospital, Marseille 13005, France; ^3^Functional and Stereotactic Neurosurgery, APHM, Timone Hospital, Marseille 13005, France; ^4^Physics Department, University of Bucharest, Bucharest, Romania; ^5^Centre de Recherche Cerveau et Cognition, Université de Toulouse, Université Paul Sabatier Toulouse, Toulouse 31052, France; ^6^Centre National de la Recherche Scientifique, CerCo (UMR5549), Toulouse 31052, France; ^7^Aix Marseille University, CNRS, LPC, Marseille, France

**Keywords:** ERP, hippocampus, human, iEEG, independent component analysis, memory recognition

## Abstract

The hippocampus is generally considered to have relatively late involvement in recognition memory, its main electrophysiological signature being between 400 and 800 ms after stimulus onset. However, most electrophysiological studies have analyzed the hippocampus as a single responsive area, selecting only a single-site signal exhibiting the strongest effect in terms of amplitude. These classical approaches may not capture all the dynamics of this structure, hindering the contribution of other hippocampal sources that are not located in the vicinity of the selected site. We combined intracerebral electroencephalogram recordings from epileptic patients with independent component analysis during a recognition memory task involving the recognition of old and new images. We identified two sources with different responses emerging from the hippocampus: a fast one (maximal amplitude at ∼250 ms) that could not be directly identified from raw recordings and a latter one, peaking at ∼400 ms. The former component presented different amplitudes between old and new items in 6 out of 10 patients. The latter component had different delays for each condition, with a faster activation (∼290 ms after stimulus onset) for recognized items. We hypothesize that both sources represent two steps of hippocampal recognition memory, the faster reflecting the input from other structures and the latter the hippocampal internal processing. Recognized images evoking early activations would facilitate neural computation in the hippocampus, accelerating memory retrieval of complementary information. Overall, our results suggest that the hippocampal activity is composed of several sources with an early activation related to recognition memory.

## Significance Statement

In the human memory circuit, the hippocampus is considered as a structure with relatively late activation, associated with the retrieval of elaborate memories. However, in most electrophysiological studies, it is characterized by the activity of a single contact, which may not represent the entire dynamics of this structure. Here, we combined intracerebral recordings with independent component analysis to separate the activity from two different neural sources generated in the hippocampus. We analyzed the responses of both sources during the recognition of old and new images. Our results reveal new dynamics associated with different neuronal sources within the hippocampus, with recognition memory occurring much faster than previously reported.

## Introduction

Recognition memory plays a crucial role in our ability to navigate and comprehend the world around us. The information retrieved is rapidly processed in the brain with behavioral responses starting ∼350 ms after stimulus presentation (Besson et al., 2012). However, the timing at which each structure is engaged/involved in this process is still obscure (Warburton and Brown, 2015).

In the hippocampus, event-related potentials (ERPs) are elicited during recognition of known faces or words, with larger amplitudes between 400 and 600 ms poststimulus (hippocampal P600) for successful item recognition (Fernández et al., 1999; Trautner et al., 2004; Barbeau et al., 2008). The timing of this response, together with the activation of other brain areas, allows a rough estimation of the memory circuit of the brain. Early potentials are generated along the whole ventral pathway (Allison et al., 1999). However, the first structure showing a modulation by the old/new status of the stimuli is the perirhinal cortex, with higher responses for known items at ∼200–300 ms, followed by the supplementary motor area, and by various frontal and parietal regions (Gonzalez et al., 2015; Despouy et al., 2020a). In this context, the hippocampus has been considered as one of the latest structures involved in recognition (Despouy et al., 2020a), with the first difference between old and new elements occurring almost 500 ms after stimulus onset. This result has suggested that the hippocampus is mainly involved in a slower process that retrieves complementary information (Despouy et al., 2020a), although early activations have also been identified for specific source memory effects (Staresina et al., 2012). A similar result was found in a recent magnetoencephalography (MEG) study, with higher amplitudes for recognition memory at ∼500 ms (López-Madrona et al., 2022). Interestingly, the responses to old and new elements were different not only in amplitude but also in delay, with faster activations for recognized items. The time delay between conditions was ∼100 and 150 ms. The origin of the MEG activity was validated using simultaneous intracerebral recordings, corresponding to the combined activity of the hippocampus and the rhinal cortex.

Despite the high spatial resolution of intracerebral recordings (stereotaxic electroencephalography, SEEG), it is extremely difficult to separate different signal sources within the hippocampus. Substructures such as CA1, CA3, and the dentate gyrus (DG) are roughly of the same size as SEEG contacts—a few millimeters. A single contact may spatially cover more than one structure, while others may not be sampled. In addition, SEEG sampling is constrained by clinical indications. Only a limited number of contacts are placed in the hippocampus and their location varies across patients. The different substructures of the hippocampus are folded one in the other, resulting in overlapped field potentials. Therefore, the hippocampus is most commonly analyzed as a single responsive area in SEEG, blurring the contribution of each substructure to the recordings (Ludowig et al., 2010; Barbeau et al., 2017).

When the activities of various structures overlap in time and space, independent component analysis (ICA) may be used to separate the time courses of the different current generators contributing to the recorded field potentials (Makarova et al., 2011; Herreras et al., 2015, 2022). ICA has been extensively used in human EEG and MEG, removing artifacts as the cardiac activity (Jung et al., 2000) or retrieving neuronal sources (Hsu et al., 2022; Velmurugan et al., 2022). In intracerebral data, the efficiency of ICA to disentangle hippocampal pathways has been well established in animal studies, where it has helped isolating the different inputs to CA1 (Benito et al., 2016; López-Madrona et al., 2020) and to the DG (Benito et al., 2014; Fernández-Ruiz et al., 2013, 2021). In humans, methods such as bipolar montages or current source density analysis are commonly used to measure the local inflow and outflow of currents in a specific location (Nicholson and Freeman, 1975; Mitzdorf, 1985). However, these methods may not recover the correct time courses of the local sources (Fernández-Ruiz and Herreras, 2013; Martín-Vázquez et al., 2013; Michelmann et al., 2018). For example, in the case of two colocalized sources (i.e., close to the same SEEG contact), the bipolar montage would measure a combination of the activities of both structures. This overlap can have critical consequences. If the sources have anticorrelated activities, one or both signals would be cancelled. Although the identification of intracerebral sources with ICA has so far been restricted to animal studies, it has been proposed as an optimal solution to remove the electrical reference (Hu et al., 2007; Whitmore and Lin, 2016), or for rereferencing intracerebral EEG data and identifying neural generators in LFP recordings (Michelmann et al., 2018). Moreover, ICA has been recently proposed as a method to localize and reconstruct remote sources nonsampled with the intracerebral electrodes (López-Madrona et al., 2023).

Using such an approach, we clearly identified for the first time different sources of the electrophysiological signal in the hippocampus during a recognition memory task. First, we applied ICA on SEEG recordings, identifying two different hippocampal current generators and disentangling their time courses from other close or distant activities. Then, we examined the spatial topography of each hippocampal component and corroborated the local origin of the sources. Finally, we characterized the dynamics of the hippocampal responses during a recognition memory task.

## Materials and Methods

### Participants

Ten patients (three females) with pharmacoresistant epilepsy volunteered for this study. They were implanted with SEEG depth electrodes along the longitudinal axis of the medial temporal lobe during presurgical evaluation. All these patients had been implanted in the hippocampus for clinical reasons (Extended Data Figure 1-1). Each electrode was placed targeting either the anterior (electrode B) or the posterior hippocampus (electrode C). The final location was determined by the neurosurgeon based on the individual anatomy of each patient. Five patients were recruited at La Timone Hospital (Marseille, France) and the other five at the Emergency University Hospital Bucharest (Romania). None of the patients presented sclerosis. For those patients recorded in Marseille, the hippocampus exhibited the stereotyped responses during an oddball task, which confirmed the correct functionality of this structure. Table 1 shows the clinical information for each patient. This research has been approved by the relevant Institutional Review Board (Comité de Protection des Personnes, Sud-Méditerranée I, ID-RCB 2012-A00644-39) and the Bucharest University ethical committee approval (CEC 23/20.04.2019). All participants signed a written informed consent form regarding this research.

**Table 1.** Clinical information of each patient

Patient	Age	Epilepsy	Language organization	Electrode(s) location
1	36	Bilateral temporomesial	Atypical bilateral	Right anterior Right posterior
2	37	Bilateral temporomesial	Left typical	Right anterior Left anterior
3	17	Left operculoinsular	Left typical	Left anterior
4	36	Right temporomesial	Left typical	Right anterior Left anterior
5	26	Bilateral extensive on heterotopia	Right atypical	Left anterior Right posterior Left posterior
6	47	Left temporomesial	Left typical	Left posterior
7	38	Right insular	Left typical	Right anterior
8	27	Left insular	Left typical	Left posterior
9	23	Right parietotemporal heterotopia	Left typical	Right anterior
10	29	Right temporo-occipital heterotopia	Left typical	Right posterior

### Experimental paradigm

Each block of the recognition memory task started with an encoding phase, during which 12 pictures were presented, one after the other, and the participant was asked to memorize them. Each picture was a simple colored drawing of a familiar item (e.g., a dog or a car) on a gray background. The picture database and precise selection criteria are described below. After a distracting video of 1 min (silent excerpts from a documentary showing birds and landscapes), the recognition phase involved a set of 24 pictures from the same database. Half of these pictures had been presented during the encoding phase, while the other 12 were new, never-presented, pictures. Participants were asked to press two different buttons if they recognized the image as having been presented earlier during encoding (“old” condition), or if the image was “new” to them. The latency of this response is the response time (RT). Stimuli presentation and response logging were controlled by the software E-prime 3.0 (Psychology Software Tools).

Each trial started with a fixation cross-presented in the center of the screen for 1,000 ms, followed by the experimental picture, presented for 1,000 ms in the encoding sub-block and for 1,500 ms in the recognition sub-block. The subsequent intertrial interval was fixed to 1,000 ms in both blocks. For each participant, a total of seven blocks were programmed to be displayed consecutively, using different images.

We selected 24 × 7 = 168 images to be used as experimental materials from the database of Duñabeitia et al. (2018). They were selected as having high name agreement (above 90%). To ensure that the observations were not driven by item-specific properties, different experimental lists were created for each participant. The items were separated into two matched groups of 84 items to serve as old and new, alternatively across patients. Across the “old” and “new” groups of items, there were roughly equal numbers of natural and artifact stimuli, with matched visual complexities; their names in French were matched for name agreement, length in syllables, and (log) lexical frequency of use [normative data from Duñabeitia et al. (2018) or New et al. (2004)]. The 84-item groups were further broken down into seven groups to be used in the different blocks, with items matched for visual complexity and (log) lexical frequency across the seven groups. All matching across picture groups was performed with the MATCH utility (van Casteren and Davis, 2006). In the encoding phases, the 12 items were presented in a random order; in the recognition phases, the items were presented in a pseudorandom order, with the constraint that there were never more than three “old” or “new” items in a row.

### SEEG recordings

SEEG recordings were performed using depth electrodes (Alcis and DIXI Medical), implanted stereotaxically (Talairach et al., 1992). The electrodes of both clinical centers had between 8 and 18 contacts per electrode, a diameter of 0.8 mm, and contact length of 2 mm and were separated from each other by 1.5 mm. We implanted between 89 and 223 SEEG contacts per patient (total contacts recorded, 1,590; mean of 159 contacts per patient, SD ± 55). A scalp electrode placed at the Fz location was used as reference for the recordings performed in Marseille, while the recordings in Bucharest used an intracranial contact located in the white matter, exhibiting the lowest activity. To determine the exact location of each electrode and contact, a postimplantation CT scan fusion with the preoperative MRI was performed for each patient. SEEG signal from Marseille center was recorded on a digital system (Natus Medical Incorporated) with sampling at 1,024 Hz or more with 16-bit resolution, a hardware high-pass filter (cutoff = 0.16 Hz), and an antialiasing low-pass filter (cutoff = 340 Hz). Data from the Bucharest hospital were recorded with a Quantum 128 system (Natus Neuro), sampling rate at 4,096 Hz, 24-bit resolution, and a hardware high-pass filter at 0.08 Hz. To homogenize both datasets, we applied digital high-pass and low-pass filters at 0.5 and 256 Hz, respectively, and down-sampled the data to 1,024 Hz. We inspected the data for interictal epileptiform discharges and other artifacts and manually removed those segments.

### ICA

Because this work is focused on the analysis of hippocampal sources, we selected for ICA only the electrodes targeting this structure. ICA was run on the continuous traces of each electrode of each patient separately. We thus analyzed signals collected using five electrodes (*N* = 15) implanted in 10 patients, having a total of 189 contacts (mean of 12 contacts per electrode, SD ± 2).

ICA aims to solve the “cocktail party” problem by separating *N* statistically independent sources that have been mixed in *M* recording contacts. It is a blind source separation methodology, as the spatial distribution and time courses of the sources are unknown. To identify the sources, ICA assumes that they are immobile in space, that is, that the proportional contribution of each source to every contact is the same throughout the recording session. Each recorded signal $u_{m}(t)$ is thus modeled as the sum of N independent sources $\left(s_{n}(t)\right)$ multiplied by a constant factor $\left(V_{mn}\right)$:

(1) $$u_{m}(t)=\overset{N}{\underset{n=1}{\sum }}\;V_{mn}s_{n}(t),m=1,2,\ldots ,M,$$

where $u_{m}(t)$ is the SEEG data, $V_{mn}$ the ICA weights or spatial distribution of each source, M the number of contacts, N the number of sources, and $s_{n}(t)$ the obtained independent components (“SEEG-ICs”).

In this work, we obtained as many components as contacts per electrode (*N* = *M*), without applying any dimension reduction (Artoni et al., 2018). We applied ICA on the continuous data, seeking one mixing matrix per electrode. We used FieldTrip (Oostenveld et al., 2011) to compute ICA based on the infomax algorithm, which aims to minimize the mutual information between components (Bell and Sejnowski, 1995), implemented in EEGLAB (Delorme and Makeig, 2004). Then, we normalized the data by *z*-scoring the continuous traces of each component to facilitate the comparison across subjects. Although only some of the SEEG-ICs were putative neuronal sources, we did not discard any component at this point.

### Analysis of event-related potentials

We segmented the continuous dataset into trials with a duration of 1 s from the stimulus onset. Only trials associated with the correct behavioral response were considered in the analysis. For each SEEG-IC, we checked if they had a significant ERP in both the “old” and the “new” conditions. To do so, we tested if each time point across trials was significantly different from zero with a *t* test, obtaining a *t*- and *p*-values for each time point. Then, we corrected these tests for multiple comparisons using a local false discovery rate (LFDR; Benjamini and Heller, 2007) on the *t*-values with a threshold of 0.2 (Pizzo et al., 2019). LFDR assumes that the distribution of *t*-values is Gaussian, considering as significant those values that stand out from the distribution. To have a better estimation of the distribution, we grouped all the *t*-values across components of each electrode, obtaining a single threshold per electrode. To remove artifactual single points, that is, single data points that were significant but the anterior and posterior samples were not, we selected only those points during the first second after the stimulus, and we imposed a minimum number of consecutive significant time samples (10 ms in this work).

To assess if the responses of the components differed in amplitude during “old” versus “new” trials, we repeated a similar analysis across conditions. For each component and time point, we performed a *t* test across trials between the amplitudes of the ERP in the old and new conditions. Then, we corrected the *t*-values for multiple comparisons using LFDR on the *t*-values of each dataset (i.e., all the components of one electrode). In this way, we identified statistical differences at the single electrode level.

### Referential and bipolar montages

To compare the results using ICA with traditional approaches to record local signal sources and sinks, we have analyzed the contacts within the hippocampus using a referential and a bipolar montage (Hirsch and Brenner, 2010). The ERPs were analyzed following the same approach as for the IC-SEEGs (see above, Analysis of event-related potentials). As several contacts may be placed within this structure, potentially recording different local activities, we followed two different criteria for contact selection. The first criterion was based on the amplitude of the response, selecting the contact with maximal amplitude for “old” images. In the second criterion, we focused on the temporal activation, selecting the contact showing the earliest activation, determined as the first time point with an amplitude significantly different from zero.

### Response without prediction

Some factors of our task design could lead to some implicit task structure learning, for example, the fixed intertrial interval, the same number of “old” and “new” items per block, or the fact that no more than three consecutive elements of the same condition could be presented. Thus, it is theoretically possible that this trial prediction is reflected in the hippocampal response. To check whether the SEEG-ICs presented a significant activation unrelated to the task predictability, we computed the ERPs using only the first trial of each block, that is, images that did not immediately precede another trial. Only for this analysis, we included trials from the encoding part of the task to increase the number of trials. A total of 14 trials were selected per SEEG-IC, seven from the encoding sub-block and seven from the recognition one.

### Identification of hippocampal components

We focused on the putative hippocampal SEEG-ICs that were responding to the protocol. To identify these components from all the obtained sources, we first selected the signals with a significant ERP during both old and new trials. Then, we considered only the components with a local spatial distribution that was maximal in the hippocampus, that is, those for which the ICA weights present a strong decay across contacts. To estimate this decay, we computed the number of contacts with an ICA weight higher than half of the maximal weight for each SEEG-IC. Therefore, we considered an SEEG-IC as locally generated in the hippocampus if the ICA weights outside this structure had a decay of, at least, half of the maximum weight.

After visual inspection, we identified two components that had a similar ERP pattern across patients. We labeled these components “Hc250-IC” and “Hc600-IC” based on the latencies of their responses. We only considered the time courses of Hc250-IC and Hc600-IC for further analysis. As ICA does not ensure the correct polarity and amplitude of the sources, we reversed the components when needed to match the same polarity across electrodes.

### Spatial distribution across recording sites

To compare the spatial topographies of components Hc250-IC and Hc600-IC, we selected the electrodes where both components were identified and measured the distance between the peaks of the ICA weights in terms of number of contacts. If the peak of Hc250-IC weights was located in a deeper contact than Hc600-IC, the distance was considered as negative, and positive if the contact was in a more lateral location. Then, we compared with a *t* test whether this difference was significantly different from zero.

To test whether other brain regions were contributing to the SEEG-ICs components, we repeated the ICA now including all the recording sites in each patient. The computation of a single ICA for all the recordings may affect the resultant time courses. Low variance hippocampal components may not be singled out, as the addition of many signals far from the hippocampus decreases the relative contribution of this source to the whole dataset. Moreover, the number of contacts and locations strongly differs across patients, hindering the intersubject comparison. Therefore, we performed the analysis in an iterative manner, including only one additional contact at a time and evaluating its contribution to the SEEG-ICs. At each iteration, we computed a new ICA on the combination of the original electrode (i.e., the electrode targeting the hippocampus) and one extra contact. We then computed the zero-lag correlation between the original SEEG-IC signal (only the hippocampal electrode) and the new SEEG-ICs (combination of the hippocampal electrode and an additional contact), selecting the component with the highest correlation. This component would represent the same neuronal source in both datasets. Then, we estimated the relative contribution of the additional contact to the component as the ratio between the ICA weights at the new location divided by the highest ICA weight. A value close to 1 would imply that the additional contact strongly contributes to the SEEG-IC, suggesting that the neuronal source is nearby, while a value close to 0 would indicate that the additional contact is relatively far from the source. This allows the analysis of the approximated spatial distribution of the components on the whole sampled brain while minimizing the impact of including more contacts on the estimation of source time courses.

### Group analysis of event-related potentials

In order to test for a significative response at the group level, either for SEEG-ICs or for referential and bipolar montages, we performed a nonparametric permutation test corrected with cluster-based statistics (Cohen, 2014). We computed the averaged ERP for each electrode and condition. Then, for each time point, we computed a *t* test against zero between the ERP values across electrodes in each condition. We kept the *t*-values of those points with a *p*-value <0.05. These are the uncorrected values of significance. To correct for multiple comparisons, we selected clusters of significance, that is, group of consecutive time points with a significant *p*-value. We assigned to each cluster the sum of the *t*-values inside the cluster (either positive or negative). We computed *N* = 2,000 surrogate datasets, by randomly shifting the starting time of the ERPs of each SEEG-IC. We selected circular ERPs of 2 s, including 500 ms of baseline. Thus, for each surrogated ERP, we chose a random value between −500 and 1,500 ms around the stimulus, which was considered as the starting point, and shifted it accordingly. This way, the ERP signals remained the same, but the temporal alignment between ERPs was broken. We repeated the cluster procedure for each surrogate, keeping both the clusters with maximal and minimal *t*-value at each iteration. Any significance found in these surrogates would be by chance. Finally, we tested whether the *t*-values of our original clusters were significantly higher than the maximal *t*-values of the surrogate analysis for positive effects or lower than the minimum for negative effects. The threshold of significance was set at the 97.5 percentile of the distribution of surrogate values (*p*-value <0.025). The same procedure was followed to compare the amplitudes of the responses across conditions. In this case, the *t* test was computed between conditions instead of against zero. To compare whether the responses were similar between hemispheres or between the anterior and posterior hippocampus, we repeated the same approach but comparing each component and condition between groups (left vs right or anterior vs posterior).

### Raster plots

We analyzed the trial-to-trial variability of the responses using raster plots. We selected all trials across electrodes and conditions and sorted them based on the RT to the stimulus. Then, we stacked them in a single matrix with dimensions time × number of trials. To assess whether the ERP was related with the patients’ response times, we correlated the latency of the ERP onset with the RT. First, we created supertrials of 50 trials pooled across electrodes and conditions to improve the signal-to-noise ratio (Hebart et al., 2018; Despouy et al., 2020a). We tested supertrials of different sizes (in numbers of trials) without observing noteworthy differences in the main result. The onset latency of each super trial was estimated using the median rule, that is, as the first time point with an amplitude higher than the median of the baseline plus 2.3 times the interquartile range (Letham and Raij, 2011). Finally, a Pearson’s correlation was applied between the onset latency and the averaged response time of each supertrial.

### Detection of slope change points

To better characterize the dynamics of the Hc600-IC response, we modeled the averaged ERP for each electrode and condition as a combination of multiple linear segments. The locations of the intersections between segments were automatically selected using the MATLAB (MathWorks) function findchangepts. This function identifies the points where the mean and the slope of the signal change most abruptly (Haynes et al., 2017). The total number of sections was given by a parametric threshold that imposes the minimum required improvement in the residual error for each change point. The residuals are strongly related with the signal-to-noise of the ERP, which was different for each electrode. Thus, we manually adjusted the threshold value between 0.5 and 3 for each ERP, until the response was clearly modeled with a minimum number of change points. The same value was used in both conditions. We determined the *t*2 value as the change point with maximum amplitude between 200 and 600 ms. Then, the *t*1 latency was selected as the first change point with a local minimum in amplitude before *t*2.

## Results

### Behavioral performance

A recognition memory task, where patients were asked to differentiate between new items and images that were presented before, was performed by 10 patients with drug-refractory epilepsy. The performance across subjects was relatively high (*d′* between 2.02 and 3.4; median = 2.64), with a hit rate (correct “old” responses) between 0.88 and 0.97 (median = 0.94) and a false alarm rate (incorrect “new” responses) between 0.05 and 0.27 (median = 0.12). After removing artifacts and incorrect responses, between 61 and 80 “old” trials (median = 70) and between 62 and 78 “new” trials (median = 71) were retained per patient for further analysis.

### Segregation of SEEG time courses into current generators

**Figure 1.**
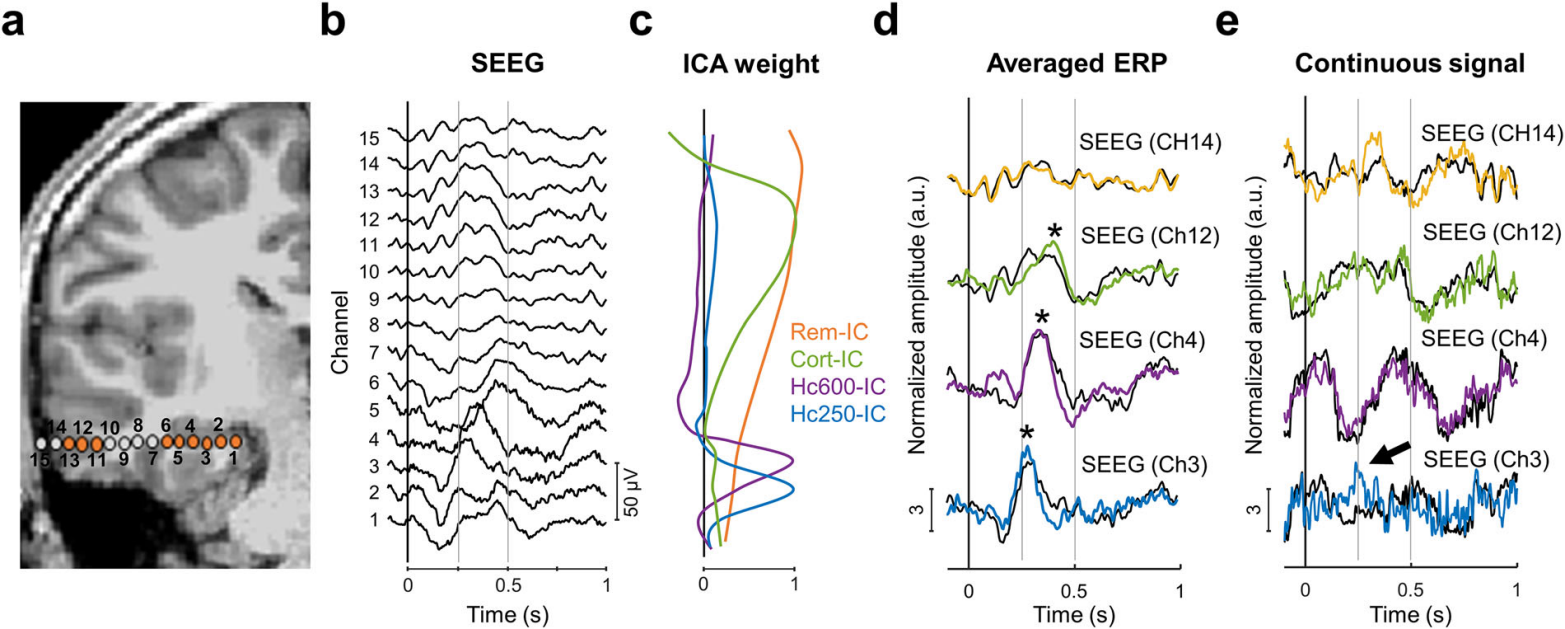
Separation of brain sources in SEEG with ICA in one patient. ***a***, MRI (3D T1) with reconstruction of SEEG electrode for Patient 1. The points represent the location of each recording site. In orange, channels with a significant ERP for “old” (i.e., previously seen) items. See Extended Data Figure 1-1 for the location of all the electrodes and Extended Data Figure 1-2 for another representative patient. ***b***, Averaged ERP for “old” items at each recording site (referential montage). ***c***, Spatial profile of the SEEG-ICs across the electrode, representing the contribution of the SEEG-ICs to each contact. ***d***, Averaged ERP of each SEEG-IC for “old” responses (color-coded traces; *p < 0.05, *t* test across trials against zero) superimposed with the SEEG channel (panel ***b***) with maximal contribution from each SEEG-IC (black traces). ***e***, Example of single-trial response for SEEG-IC and SEEG at the peak of the ICA weight. The arrow indicates a response to the stimulus that can be identified in SEEG-IC traces, but not in the original SEEG data.

We performed electrophysiological recordings from the hippocampus in 10 patients during a recognition memory task (Fig. 1*a*; see Materials and Methods). We selected a total of 15 electrodes (*N* = 15) implanted in this structure among all participants. For each electrode, we applied the ICA source separation technique to segregate the recordings into the main sources contributing to the SEEG activity. The general pipeline and the comparison between ICA and raw SEEG for one patient are depicted in Figure 1. Four different types of independent components (SEEG-ICs) were identified in most of the electrodes. The spatial profile of each component (Fig. 1*c*) reflects its contribution to each SEEG contact, allowing to infer the location of the component's source. The first component (labeled “Cort-IC”) was present in 13 out of 15 electrodes, and it was mainly visible on the lateral contacts of the electrode with a progressive reduction toward deeper structures (between three and seven contacts with an ICA weight higher than half of the maximum, median: 4) and presumably reflects a source located in the lateral temporal cortex. Two components had their maximal contribution inside the hippocampus and were labeled as “Hc250-IC” and “Hc600-IC” based on the latency of the ERP (Fig. 1*d*). The former had its highest amplitude at ∼250 ms, while the latter, with a peak at ∼400 ms, may be related with the “hippocampal P600” commonly identified in visual recognition tasks (Trautner et al., 2004; Barbeau et al., 2017). Both components presented a narrow spatial profile. They had between one and three contacts with an ICA weight higher than half of the maximum (median: 1), all of them within the hippocampus, strongly suggesting that these components represent local sources inside this structure. The fourth component probably represents a remote source far from the electrode (“Rem-IC”). It was present in 13 out of 15 electrodes, with similar contribution to all the contacts (between 4 and 15 contacts with an ICA weight higher than half of the maximum, median: 9).

10.1523/ENEURO.0183-23.2023.f1-1 Figure 1-1 **Location of all the electrodes** Orange dots represent the channel location for each electrode in MNI space. The number indicates the patient. The shaded area depicts the hippocampus. Download Figure 1-1, TIF file.

**Figure 2.**
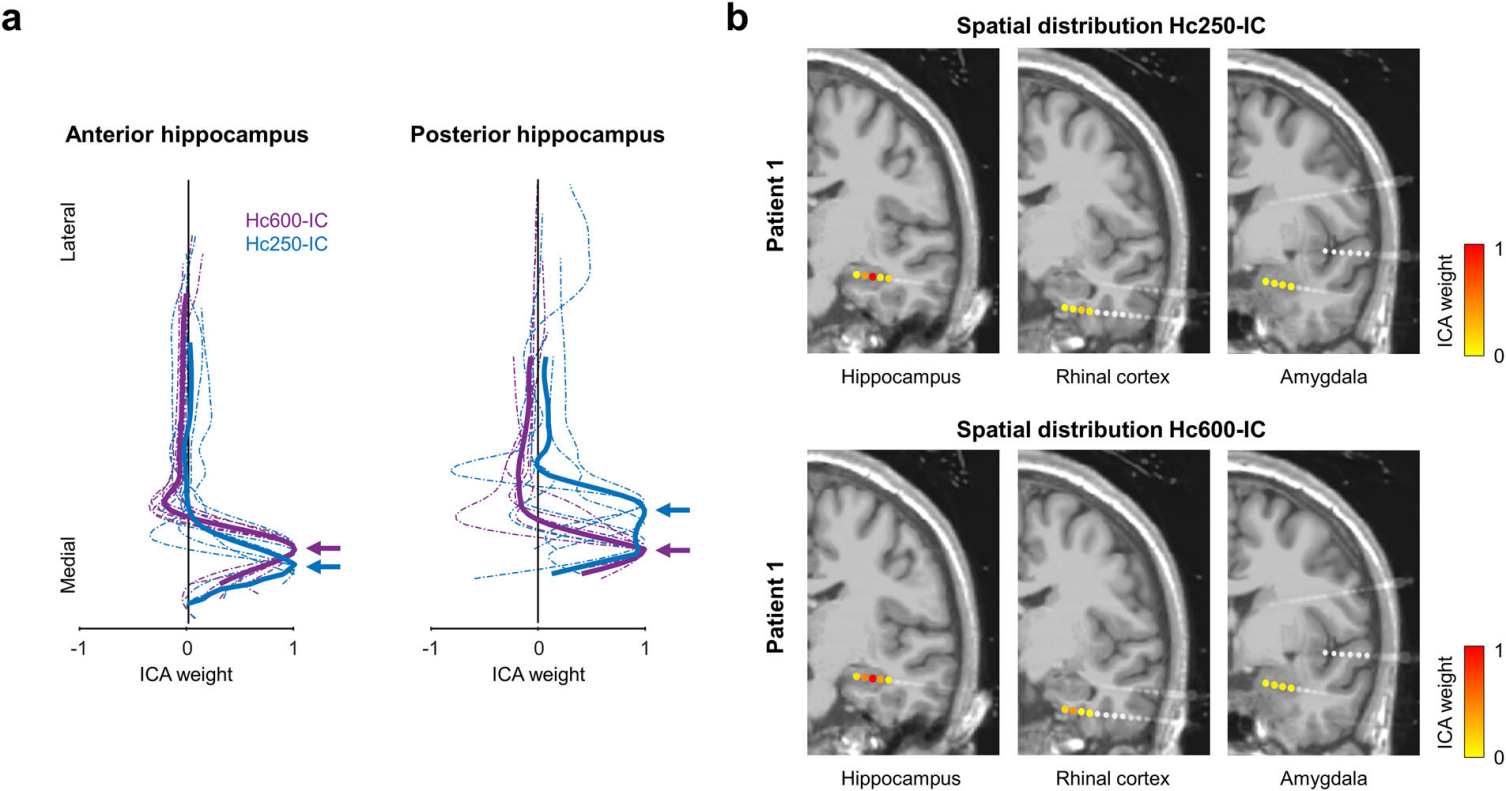
Two hippocampal sources during recognition memory. ***a***, Spatial profile of the two hippocampal SEEG-ICs across electrodes located in the anterior or posterior hippocampus. Dashed and solid traces represent the individual electrodes and their averaged value, respectively. Electrodes from different patients have been aligned based on the peak of Hc600-IC. Arrows indicate the location of the maximal value for the averaged profiles. ***b***, MRI (3D T1) and reconstruction of SEEG electrodes for Patient 1, where the rhinal cortex and the amygdala were both sampled with SEEG. The color of the contact represents the spatial distribution of Hc250-IC (top) and Hc600-IC (bottom). The maximal contribution is in the hippocampus, with low values in all the other contacts.

10.1523/ENEURO.0183-23.2023.f1-2 Figure 1-2 **Separation of colocalized hippocampal sources in SEEG with ICA**
a) MRI (3D T1) with reconstruction of SEEG electrode for patient 3. The location of each recording site is represented with orange points.b) Averaged ERP for old responses at each recording site (referential montage).c) Spatial profile of the SEEG-ICs across the electrode. Both components are maximal at the same contact, but their spatial profiles differ.d) Averaged ERP for old responses of SEEG-ICs (color-coded traces) superimposed with the referential montage at the location of maximal contribution from each SEEG-IC (channel 3, black traces. While Hc600-IC correlates with the SEEG response, the early response from Hc250-IC cannot be appreciated in the raw SEEG.e) Same as panel d, but with a bipolar montage for SEEG. As both sources are colocalized close to channel 3, the local currents obtained with the bipolar montage represent the main current generator (Hc600-IC), which hides the activity from Hc250-IC. Note that ICA was always computed on the referential montage. Download Figure 1-2, TIF file.

The spatial profiles in Figure 1*c* show that the contribution of the different sources to the SEEG signals present an overlap, indicating that each contact contains information from several components simultaneously. This is most noticeable in the hippocampus, where a single contact records activities from Rem-IC, Hc250-IC, and Hc600-IC (Fig. 1*c*, channel 3). ICA separates the time courses associated with the sources, removing the contribution from other areas. For sources that are sufficiently separated in space, differences between SEEG-IC and SEEG may seem minor in the averaged ERPs (Fig. 1*d*). However, these differences are remarkable in the continuous traces, where a response to the stimulus that is apparent in the SEEG-IC is hidden in the SEEG (Fig. 1*e*, arrow). This contrast between SEEG-IC and SEEG is most prominent when the sources overlapped in space (Extended Data Figure 1-2). In this case, the activity of each source cannot be inferred from raw SEEG recordings (not even with bipolar montages; Extended Data Figure 1-2), and source separation methods are required to disentangle the time courses of the different components (Michelmann et al., 2018).

### Location and modulation of hippocampal sources

The number and responses of cortical and remote sources retrieved with ICA varied across patients, as it strongly depends on the specific implantation scheme and the number of contacts per electrode. Thus, we focused our study on the two main hippocampal sources (Hc250-IC and Hc600-IC), which were relatively stable across patients. Hc250-IC was present in 12 electrodes, and its contribution to the dataset was small (explained variance across electrodes, 4.5%, SD ± 3.2%). All patients had, at least, one electrode with the Hc250-IC source. Hc600-IC was identified in 13 of the 15 electrodes included in the analysis (9 out of 10 patients) and represented an important contribution to the total variance of the data (mean explained variance, 29.2%; standard deviation, SD ± 14.2%). These components had a similar spatial distribution across patients; their maximal contribution was in contacts located inside the hippocampus, with little contribution from other contacts (Fig. 2*a*). Within the hippocampus, the spatial profiles of both components presented were slightly different. Hc250-IC was recorded in contacts deeper than Hc600-IC in the electrodes placed in the anterior hippocampus (the peaks of the ICA weights were separated by 0–3 contacts toward the same direction; median, 1; *t* test against zero, *p* = 0.025; *t* = 3.16; df = 5; Fig. 2*a*, left). Intriguingly, this location was reversed in electrodes placed in the posterior hippocampus, where Hc250-IC appeared preferentially in more superficial sites (peaks separated by 0–1 contacts toward the same direction; median, 1; *t* test against zero, *p* = 0.037; *t* = 3.09; df = 4; Fig. 2*a*, right). This reversal may reflect rostral to caudal anatomical differences of the human hippocampus, well stratified in its anterior part and with complex folds of its substructures in more posterior sections (Andersen et al., 2006). Conversely, we did not observe differences neither in the spatial (*t* test between peak distances in the left and right electrodes, *p* = 0.90; *t* = 0.13; df = 9) nor in the ERP between electrodes implanted in the left and right hemispheres (*p* > 0.05, permutation test between old responses in the left vs right components, both for Hc250-IC and Hc600-IC).

Although the spatial distributions of the SEEG-ICs presented a clear peak inside the hippocampus, this does not completely exclude the possibility that they reflect sources from a nearby region. To further test whether the sources were truly located in the hippocampus, we repeated the ICA now including all the contacts available in each patient in an iterative manner (see Materials and Methods). For both Hc250-IC and Hc600-IC, the spatial distribution was in all cases maximal in the contacts within the hippocampus, followed by the amygdala (ICA weights between 0.006 and 0.27 times the peak value, median: 0.16) and the rhinal cortex (ICA weights in contacts within the rhinal cortex were between 0.02 and 0.18 times the peak value in the hippocampus, median: 0.042), but with negligible contributions (Fig. 2*b*). This result reinforces the interpretation of the hippocampus as the origin of the components.

**Figure 3.**
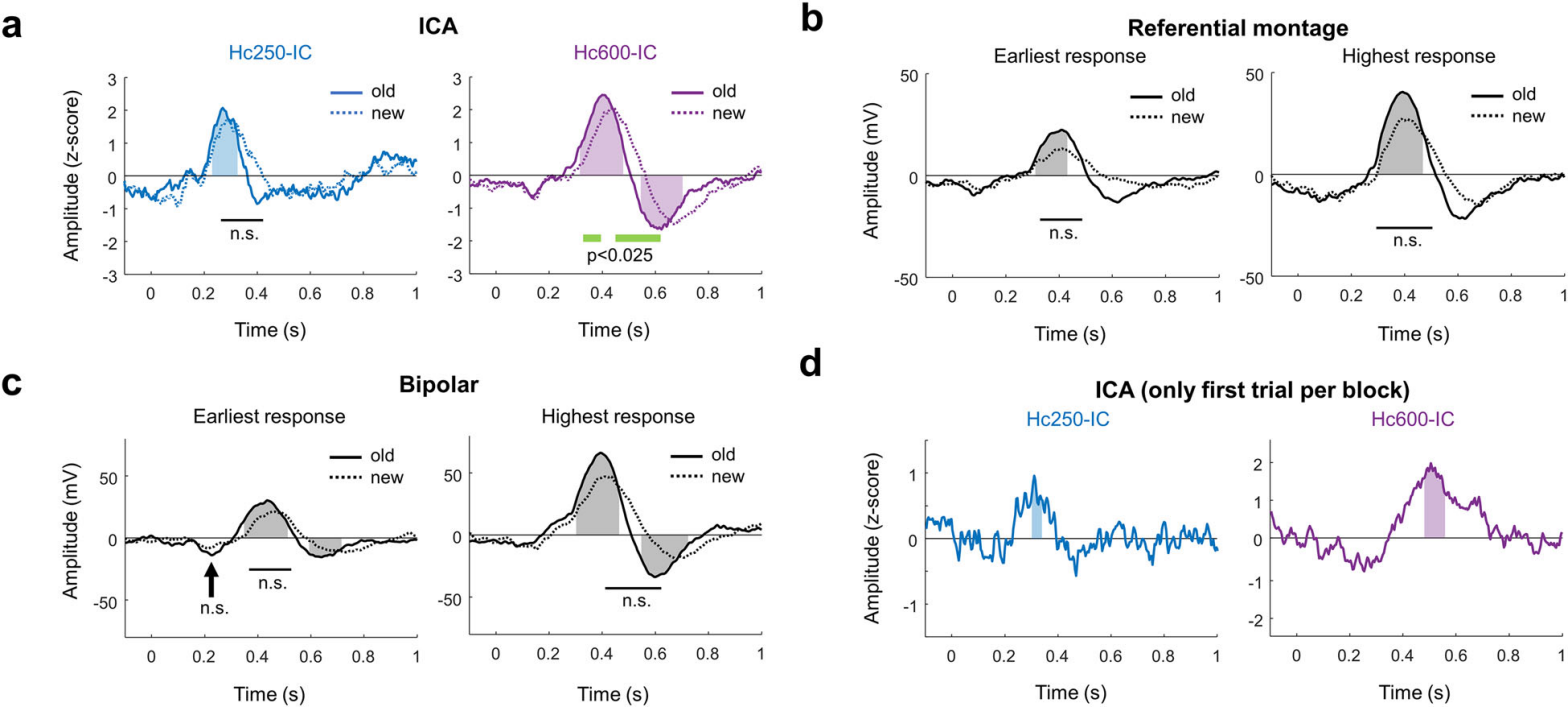
Group-level analysis of hippocampal responses. ***a***, Grand average IC-ERP across electrodes contrasting “old” (solid traces) and “new” (dashed traces) items for Hc250-IC and Hc600-IC. For all panels, shaded areas illustrate the intervals where the response to “old” trials is significantly different from zero, while the green line indicates the interval with differences between conditions (*p* < 0.05, permutation test; NS, not significant). See Extended Data Figures 3-1 and 3-2 for single-case analysis. ***b***, Grand average ERP using a referential montage. For panels ***b*** and ***c***: on the left, the contacts in the hippocampus with the earliest activation (first time sample significantly different from zero) were selected for each electrode. On the right, the contacts with the highest response in amplitude to “old” images were chosen. ***c***, Grand average ERP when selecting a single pair of contacts per electrode with a bipolar configuration. ***d***, Grand average between SEEG-ICs without predictive effects. For each component, only the first trial of each block was selected, that is, images that did not immediately precede a previous trial.

The temporal profile of the components presented similar ERPs across electrodes (Fig. 3*a*). There were no differences between components in the anterior and posterior hippocampus (*p* > 0.05, permutation test between old responses in the left and right components, both for Hc250-IC and Hc600-IC). The earliest response was in Hc250-IC, with a single peak at ∼260 ms (mean, 257 ± 42 ms across patients) poststimulus during “old” items (Fig. 3*a*, left) that was significantly different from zero in all electrodes (*t* test across trials against zero, corrected with LFDR). The response to “new” items presented a similar timing as to recognized elements, but with significant differences in amplitude between both conditions in 6 out of 12 electrodes (old/new contrast; Extended Data Figure 3-1; *t* test across trials between conditions, corrected with LFDR). However, there were no differences at the group level (*p* > 0.05, permutation test). The response of Hc600-IC elicited by old items was characterized by two peaks of opposite polarity at 405 and 610 ms poststimulus onset (Fig. 3*a*, right), similar to the standard hippocampal response in other memory tasks (Trautner et al., 2004; Barbeau et al., 2008, 2017). Comparing the responses between conditions, Hc600-IC presented different amplitudes for “old” and “new” conditions ∼400 and 500 ms at the group level (Fig. 3*a*; *p* < 0.05, permutation test). This difference was also present at the single electrode level in 12 out of 13 cases (Extended Data Figure 3-2).

10.1523/ENEURO.0183-23.2023.f3-1 Figure 3-1 **Single-case comparison between old and new responses of Hc250-IC** Each plot represents the averaged ERP of Hc250-IC during old (solid traces) and new (dashed traces) trials for each electrode. Hashes represent patient number and stars indicate significant differences in amplitude between conditions (* p < 0.05, t-test across trials corrected with LFDR). Only 6 out of 12 electrodes presented a modulation to the memory protocol. In three cases, this difference was due to higher amplitudes after the presentation of old images at early latencies (electrodes 5, 10 and 12). In the other three electrodes, the responses were different, with the responses to the new images standing high during a longer period (electrodes 2, 4 and 7). Download Figure 3-1, TIF file.

10.1523/ENEURO.0183-23.2023.f3-2 Figure 3-2 **Single-case comparison between old and new responses of Hc600-IC** Each plot represents the averaged ERP of Hc600-IC during old (solid traces) and new (dashed traces) trials for each electrode. Hashes represent patient number and stars indicate significant differences in amplitude between conditions (* p < 0.05, t-test across trials corrected with LFDR). A total of 12 out of 13 electrodes presented a modulation to the memory protocol. Download Figure 3-2, TIF file.

We then examined the ERPs using two traditional approaches: referential and bipolar montages. For each electrode, we selected the contact in the hippocampus with the earliest significant response, or the contact with the highest response in amplitude to “old” images (Fig. 3*b,c*). In 6 out of 15 electrodes, both conditions were fulfilled by the same contact in the referential montage (7 out of 15 in bipolar), hindering the direct identification of an early response different from the main P600 activity. Although both strategies and montages revealed significant responses to “old” items at ∼400 and 600 ms (*p* < 0.05, permutation test), similar to those identified in Hc600-IC, we did not identify any significant response at ∼250 ms (as in Hc250-IC) at the group level (*p* > 0.05, permutation test). These results suggest that, while the main dynamics can be identified with classical approaches, close sources cannot be easily separated, due to mixing of their activities, making the detection of responses with low signal-to-noise ratio problematic.

To test whether the SEEG-ICs were exclusively reflecting the predictability of the task (fixed intertrial interval), we measured the ERP of each component by selecting only those trials that could not been predicted (i.e., the first trial of each block). Both components exhibited a significant response at the group level (Fig. 3*d*; *p* < 0.05, permutation test), suggesting that the SEEG-ICs were active even in the absence of any temporal anticipation to the trials.

### Analysis of hippocampal dynamics

**Figure 4.**
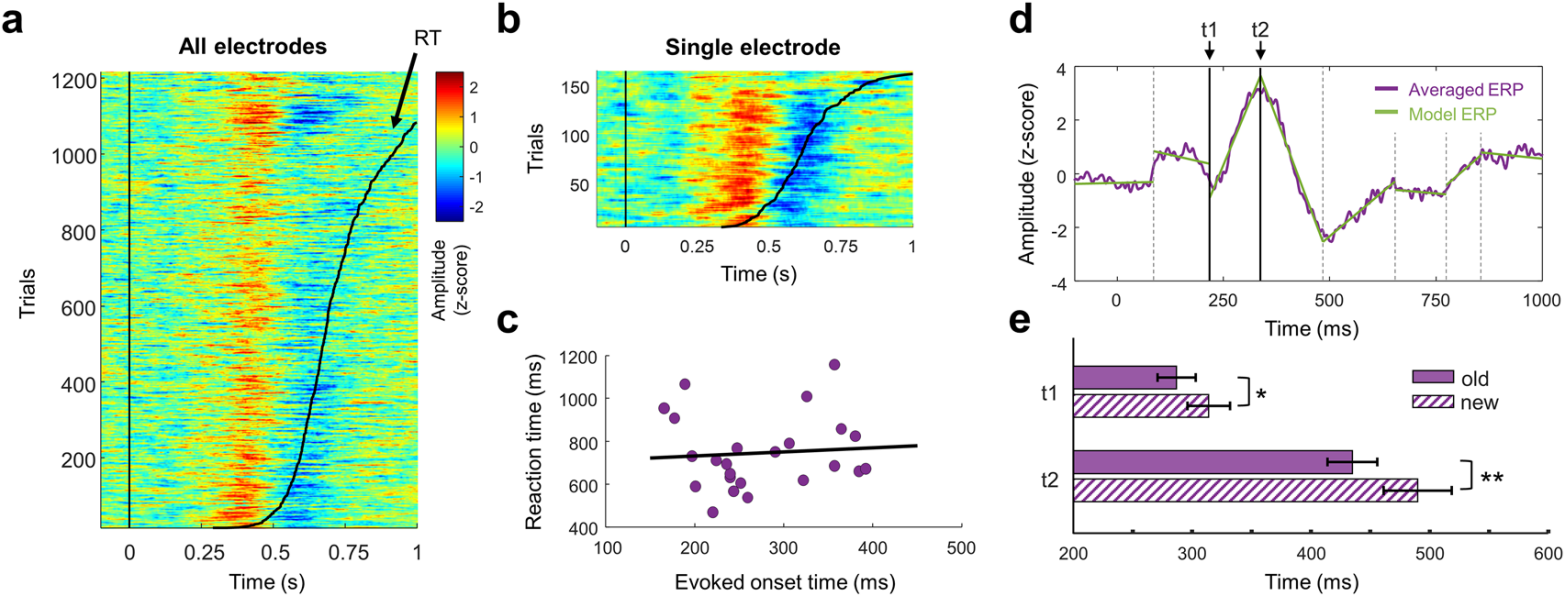
Comparison of Hc600-IC for old and new responses. ***a***, Raster plot with single-trial ERP for old and new conditions across electrodes. Trials from all patients were ordered based on the response time (black curve). ***b***, Raster plot for Subject #5. ***c***, Correlation between the evoked onset time and the RT. Each dot represents data grouped across trials (supertrial, see Materials and Methods). ***d***, Example of change point detection in the averaged ERP for old responses of one electrode. The ERP (purple trace) is modeled by several linear segments (green lines). The intersections between segments (vertical black lines) represent the time points with the highest change in mean and slope. Two change points are selected: *t*1 where the amplitude starts rising and *t*2 where the amplitude is maximal. ***e***, Comparison between *t*1 and *t*2 latencies across electrodes (*/**p < 0.05/0.01, paired *t* test between conditions, *N* = 13).

The results of Figure 3*a* and Extended Data Figure 3-2 suggested that the temporal dynamics of Hc600-IC were different between conditions, with an earlier activation time associated with the familiar items. Therefore, we further explored the modulation of this component. First, to confirm that the differences in responses between conditions were related to the memory paradigm, we selected all the single trials for both “old” and “new” conditions and reordered them based on the behavioral RT, either for all the electrodes together (Fig. 4*a*) or for each electrode separately (Fig. 4*b*). To quantify whether the hippocampal response was related to the RT, we grouped the data into supertrials and computed the correlation between the evoked onset time and the averaged RT of each supertrial (see Materials and Methods), with no significant relation between the timing of the Hc600-IC response and the RT (Fig. 4*c*; correlation test, *N* = 24; supertrials, *r*^2^ = 0.006; *p* = 0.71). This null result is in good agreement with previous studies, where the RT did relate to the latency of the response of the perirhinal cortex and the motor cortex, but had no discernable effect on the hippocampal response (Despouy et al., 2020a).

Second, to better analyze the properties of the temporal response, we modeled the averaged ERP of each condition and electrode as a combination of multiple linear sections (Fig. 4*d*). The juncture between sections was statistically defined as the time points where the mean and the slope of the signal changed most abruptly (see Materials and Methods). We tested if the timing of these change points differed between conditions. Two points of the ERP were selected for each electrode: the latency at which amplitude starts to increase (*t*1) and the latency of the maximal response (*t*2). Responses to “old” images exhibited significantly earlier latencies at both *t*1 (mean latency ± standard error of the mean; SEM, 287 ± 16.1 ms and 314 ± 17.9 ms for old and new responses, respectively; paired *t* test across electrodes, *p* = 0.011; *t* = −2.98; df = 12) and *t*2 (mean latency ± SEM, 431 ± 21 ms and 490 ± 28.7 ms for old and new responses, respectively; paired *t* test across electrodes, *p* = 0.007; *t* = −3.25; df = 12; Fig. 4*e*). As some patients had more than one electrode implanted, which may bias the results, we repeated the analysis averaging the response time across electrodes in patients with higher spatial coverage, without affecting the results of *t*1 (294 ± 21.4 ms and 318 ± 22.7 ms for old and new responses, respectively; paired *t* test between conditions across patients, *p* = 0.028; *t* = −2.68; df = 8) and *t*2 (439 ± 27.6 ms and 494 ± 33.8 ms for old and new responses, respectively; paired *t* test between conditions across patients, *p* = 0.003; *t* = −4.2; df = 8). This implies that the hippocampal processes related to recognition memory start as soon as 290 ms poststimulus onset, ∼120 ms before the latency of its maximal response.

## Discussion

We described two different hippocampal sources recorded during a recognition memory task in humans. Patients with intracerebral electrodes implanted for focal drug-resistant epilepsy monitoring were asked to classify images as “old” or “new.” Using ICA on the SEEG recordings, we disentangled two hippocampal components (i.e., the spatial distribution maximum was inside the hippocampus) from other cortical and remote sources. The first source (Hc250-IC) had a low contribution to the total variance and presented an early response to the stimulus, with significant differences between memory conditions in 6 out of 12 electrodes. The second one (Hc600-IC) had a higher contribution to the total variance and was modulated by the memory condition in all cases, with faster responses for known images compared with “new” items. The earliest difference was found in Hc600-IC at 290 ms, confirming that the hippocampus has an early involvement in recognition memory (Staresina et al., 2012).

### Identification of two hippocampal sources in SEEG

In this work, we have identified two different hippocampal neural generators and, thanks to the use of ICA, separated their corresponding electrophysiological activity. The presence of several memory-related sources in the hippocampal formation has been previously reported (Ludowig et al., 2010; Barbeau et al., 2017). Ludowig et al. (2010), describing two different generators, in the subiculum and in the posterior hippocampus, during an oddball task. These structures presented a similar ERP with the characteristic P300, but with different voltage gradients along the contacts, which indicated a local origin of the activities. It is unlikely that they are the components of our study, which we found in both the anterior and posterior parts of the hippocampus. Moreover, Hc250-IC and Hc600-IC had very different response time, in contrast with the generators identified by Ludowig and colleagues.

Previous studies have suggested the presence of an early response (∼250 ms) from the hippocampus, modulated by stimuli repetition (James et al., 2009; Nahum et al., 2011; Raynal et al., 2020). This response would coincide in time with the one in Hc250-IC. However, they are unlikely to be generated by the same source. While in these studies the activity was related to memory encoding, we did not observe such changes for new elements. On the contrary, our results have the opposite tendency, with higher amplitudes during recognition memory (Extended Data Figure 3-1). Moreover, the variance of Hc250-IC was quite low in the intracerebral recordings (4.5%), making its fingerprint on the surface likely to be negligible on raw EEG recordings (James et al., 2009; Raynal et al., 2020).

In Barbeau et al. (2017), they observed that the evoked responses during memory and novelty detection had opposite polarities, raising the possibility of different sources for each response. It is possible that both sources were always present, but with one predominating over the other for each task. In this scenario, Hc600-IC would be the main generator modulated by recognition memory, while Hc250-IC could be related to a hippocampal source also involved in novelty detection. This would explain its faster activation (the response to novelty is believed to be faster than recognition: Norman and O’Reilly, 2003; Barbeau et al., 2017). However, the lack of a clear difference at the group level in Hc250-IC hinders this interpretation. In one of the patients of Barbeau et al. (2017), the authors described an early activation at ∼200 ms during both tasks that were not present at the group level. It is possible that this early activation corresponds to a different source within the hippocampus related to Hc250-IC, which could not be identified in most patients due to its low explained variance. Together with the absence of differences in this activation between novelty and recognition memory, this component may be reflecting a visual input to the hippocampus from other cortical structures (Sehatpour et al., 2008). Further studies combining memory and novelty tasks will address this question.

### Early recognition memory system of the hippocampus

It has been suggested that the existence of two different recognition memory systems in the brain (Despouy et al., 2020a): one fast, linked to familiarity processes, and the other slow, more related to memory recollection (Yonelinas, 2002; but see Wixted and Stretch, 2004; Gimbel and Brewer, 2011 for reports where recollection-based responses are faster than familiarity-based responses). According to this theory, the fast system would respond between 200 and 300 ms after stimulus onset, and it would involve a number of areas in the frontal, temporal, and parietal lobes, led by the perirhinal cortex (Gonzalez et al., 2015; Despouy et al., 2020a). This system may reflect our interaction with the environment, allowing us to rapidly react to any stimulus. The second system would be triggered at ∼450 ms after the stimulus, when the hippocampus and other areas of the medial temporal lobe are activated (Despouy et al., 2020a). At this stage, more elaborated memories are retrieved with additional information.

An early hippocampal response was reported in Staresina et al. (2012) at ∼250 ms for “source memory” effects, followed by an “item recognition” modulation starting at ∼800 ms. The former effect coincides in time with the Hc250-IC main response. However, as the source memory was not measured in our experiment, it could also be an early modulation of the Hc600-IC source, or even belong to a third component. The later effect (∼800 ms) during item recognition may be comparable with our old/new paradigm, with a similar response in both cases. The analysis of Hc600-IC, based on delays, suggests that the differences between item recognition and correct rejections may be much earlier than those based solely on voltage differences.

As most of previous studies have focused their analysis on the main hippocampal response, the so-called hippocampal P600 (Trautner et al., 2004; Dietl et al., 2005; Barbeau et al., 2008, 2017; Despouy et al., 2020a), it has been suggested that this structure cannot be involved in fast memory processing, which would occur much earlier. However, thanks to the use of ICA in SEEG recordings, we have found that the hippocampal dynamics during recognition memory are very complex, with at least two different generators contributing to the response. Our results, with Hc600-IC presenting the earliest differential activity at 290 ms, challenge the vision of the hippocampus as a “late” structure.

We propose that both components (Hc250-IC and Hc600-IC) represent two steps of processing. The earliest component, Hc250-IC, presents similar delays as the N240 component evoked in the EC and in other mesial structures (Barbeau et al., 2017). Thus, it may reflect the input from the EC, the main entrance pathway to the hippocampus (Fernández-Ruiz et al., 2021; López-Madrona and Canals, 2021). This activity may already contain a preidentification of known elements, performed in the perirhinal cortex (Despouy et al., 2020a). The preprocessed information from EC may trigger the internal hippocampal circuit (linked to Hc600-IC), facilitating memory recollection of those elements already recognized (Yonelinas, 2002). This neuronal facilitation would be reflected in the ERP, with earlier latencies for old items (Fig. 4*e*). A similar facilitation pattern was found in MEG during the same task (López-Madrona et al., 2022). In that work, the combined activity of the hippocampus and the rhinal cortex presented earlier latencies for old items, although the onset time of the differential activity was not measured. Our findings extend previous results with a detailed characterization of the hippocampal dynamics and further confirm the contribution of Hc600-IC to the MEG activity.

Hc250-IC presented a significant modulation to recognition memory only in 6 out of 12 electrodes. Although it is not a negligible value, it cannot confirm that this source is involved in a fast recognition memory system. Instead, it could reflect a cortical input to the hippocampus from the stimulus presentation (Sehatpour et al., 2008), or it may be related to the visual perception system (Turk-Browne, 2019). In a word recognition task, Mormann et al. (2005) found an early (∼200 ms) hippocampal phase and amplitude reset produced by an unspecific mechanism which does not distinguish between mnemonic functions. Therefore, such resetting may be captured by Hc250-IC. Another possibility is that Hc250-IC reflected the predictability of the task structure. The human hippocampus may predict the temporal organization of our task, with a fixed intertrial interval (Umbach et al., 2020; Reddy et al., 2021), or even the task structure, as no more than three consecutive old or new items were presented. While we cannot exclude an effect of the implicit structure learning (Turk-Browne et al., 2010), Hc250-IC responds to every image presentation, even on the first trial (Fig. 3*d*), suggesting that it is present even in the absence of task predictability.

Overall, our results support the role of the hippocampus in memory recollection and show that it is not a late responding structure, but it is activated relatively fast (∼290 ms) during recognition memory. This delay is within the range of the ERP responses that reflect familiarity (Brown and Aggleton, 2001; Besson et al., 2012) and the fast memory system described in Despouy et al. (2020a). However, the memory task in this work cannot differentiate between different cognitive processes, limiting any direct link between the hippocampus and familiarity. Further work is required, for example, pushing the participants to their quickest answer and exploring the hippocampus together with other areas involved in the task.

### Relationship with scalp ERP components

There are two scalp ERP memory components that have been widely studied (Rugg and Curran, 2007). The FN400, a midfrontal negative ERP observed ∼400 ms after stimulus onset, is closely associated with familiarity. When individuals encounter a previously seen stimulus, the FN400 shows reduced amplitude, without necessarily retrieving detailed contextual information. On the other hand, the late positive component (LPC), a late positive-going ERP occurring between 500 and 800 ms poststimulus, has been linked to memory recollection. This component reflects the retrieval of specific details and contextual information. While the LPC component is believed to be hippocampus-dependent (Düzel et al., 2001), this structure does not contribute to the FN400 (Hoppstädter et al., 2015).

An important question is to what extent Hc250-IC and Hc600-IC may be related to these classical scalp ERP components. Previous studies with simultaneous recordings on the same task have not identified any hippocampal response at ∼250 ms neither in EEG (Barborica et al., 2023) nor in MEG (López-Madrona et al., 2022). Therefore, it is unlikely that the Hc250-IC potential can be detected from the scalp. Together with the absence of clear amplitude differences and the midfrontal topography of the FN400, our results cannot support any major role of Hc250-IC in the scalp ERP. Otherwise, the Hc600-IC potential is visible at the surface in MEG recordings (López-Madrona et al., 2022) and may directly contribute to the LPC component, as both have similar activation time. Our results, with earlier delays for recognition memory in Hc600-IC, indicate that the hippocampus could be also indirectly involved in the generation of the FN400 by activating other areas. However, this can be also the role of the perirhinal cortex, triggering the FN400 component independently of the hippocampal activity. Further studies combining intracerebral recordings from the hippocampus and the perirhinal cortex with simultaneous scalp recordings (Barborica et al., 2023) may give us new insights in the neural mechanism of the dual process of recognition memory.

### Anatomical considerations of hippocampal current generators

The activity recorded at each contact with SEEG reflects the summation of several close and distant sources (Buzsáki et al., 2012). The main features that determine the field potential of one region are the geometry and the degree of synchronization of the current sources (Herreras, 2016). Blind source separation methods such as ICA have been proposed as solutions to recover the time courses associated with specific current generators (Herreras et al., 2015, 2022). Due to its versatility, ICA has been used to remove the reference signal in intracerebral EEG (Hu et al., 2007) and to separate neural sources in EEG (Tang et al., 2002; Onton et al., 2006), MEG (Malinowska et al., 2014; Barborica et al., 2021), and local field potentials in rats (Makarov et al., 2010; Torres et al., 2019; Fernández-Ruiz et al., 2021). In this work, we innovatively used ICA to disentangle multiple generators in SEEG (Michelmann et al., 2018).

Several hippocampal generators have been described in animal studies (Korovaichuk et al., 2010; Benito et al., 2014; López-Madrona et al., 2020; Fernández-Ruiz et al., 2021), reflecting the inputs to different layers of CA1 and the DG. These two structures present a suitable anatomy to generate electric fields. In CA1, the most dominant generators are located in the stratum radiatum, with the input from CA3 through the Schaffer collateral (Korovaichuk et al., 2010; Benito et al., 2014) and in the stratum lacunosum-moleculare (Benito et al., 2014), where are located the synaptic outputs of layer III of the EC. In the DG, the highest potential is generated by the projections from layer II of the EC to the granular cells (Korovaichuk et al., 2010; Makarova et al., 2011; Benito et al., 2014). We speculate that the current generators in these two structures might be the origin of our components, with Hc250-IC related with the input of EC to the DG and Hc600-IC reflecting the computations in CA1.

The anatomy of the hippocampus differs from rostral to caudal (Andersen et al., 2006). The anterior section presents a relative clear distribution of the different layers, with the subiculum, CA1, and CA3 in the lateral part, surrounding the DG in between (Amaral, 1999). This results in well-localized components, with similar topographies across electrodes (Fig. 2*a*, anterior hippocampus). In good agreement with our hypothesis, the spatial profiles of Hc600-IC are deeper than those of Hc250-IC, which may relate Hc250-IC with lateral areas (but see below). On the contrary, the posterior section of the hippocampus has a different anatomy, with several folds of the granular layer, forming small “dents.” This geometry impacts the spatial distribution of its current sources, with summation and cancelation of currents caused by the opposite orientation of the cells through the gyrus (Buzsáki et al., 2012). Our results reflect the complexity of this area, with a huge variety in the shape and location of the topographies of the two identified hippocampal sources across electrodes/patients (Fig. 2*a*, posterior hippocampus).

It is important to note the complexity of the hippocampal circuit to avoid simplistic interpretations. For example, the DG includes a dense inhibitory network, back projections from CA3, and several inputs from the lateral and medial EC with different information (Andersen et al., 2006). Therefore, the identified generators cannot be linked to a single pathway or process. Moreover, the location of the maximal evoked potential may not coincide with the origin of the current source (Herreras, 2016; Herreras et al., 2022). Due to the curvature of CA1, a synchronized synaptic input to the whole coronal layer (i.e., from the boundary with CA3 to the subiculum) would generate field potentials across the layer, whose sum would be maximal in the center of the curve. In this situation, an activation of CA1 could be detected inside the DG. This effect has been described in the DG, where the synaptic input of the EC was in the molecular layer, but its field potential was dominant in the hilar region (Fernández-Ruiz et al., 2013). Further information of the gradients of the field potentials across the hippocampus may help to identify the true origin of the components, for example, using microelectrodes to improve the resolution across SEEG contacts (Ulbert et al., 2001). Ultimately, a realistic computational model of the human hippocampus is necessary to understand the origin of the multiple source generators (Herreras et al., 2015).

### Limitations

One aspect that remains to be studied is the dynamics of each hippocampal component associated with incorrect responses. In this work, the experiment was designed to maximize the number of correct responses, resulting in relatively high subjects’ performance (hit rate between 88 and 97%). On average, there were <15 incorrect trials per patient across all the conditions, a low number to perform a proper performance-based analysis. A second limitation in our work is the fixed intertrial interval, which may result in anticipatory responses related to trial timing. Applying a jitter to trials is a good practice both for brain responses and statistical models. We have tested whether both components have a significant activation on the first trial of each block (Fig. 3*d*). Our results suggest that the components respond to the stimuli before the anticipation processes could intervene, although we cannot completely exclude an effect of the fixed time interval in the ERP.

It has been shown that ICA is an optimal methodology to disentangle overlapping sources with microelectrodes in rodents (Makarov et al., 2010; Herreras et al., 2022) and close and distant sources in humans (Michelmann et al., 2018; López-Madrona et al., 2023). However, its efficacy to separate close sources may be limited in macroelectrodes. We assumed that the resolution of the current macroelectrodes allows capturing the differences in spatial profiles between relatively large substructures, such as the hippocampus. Microscale local field potentials provided with the new generation of microelectrodes (Despouy et al., 2020b) would be interesting for gaining access to a finer scale or single-unit activity, but this would require a larger spatial sampling, (e.g., Utah Array-like, Cody et al., 2018) than the typical sampling of current electrodes.

ICA does not ensure the correct polarity and amplitude of the sources. It is possible to project back the IC activity to the contacts by multiplying a single SEEG-IC signal by its ICA weights. By doing so, we can measure the amplitude (in volts) generated by one single source at each recording site (also referred to as virtual SEEG). The amplitude of the virtual SEEGs would be equivalent to those obtained with a referential montage. However, it should be noted that the true amplitude of the source (both with ICA or standard approaches) depends on other technical factors as the size of the contacts or the DC component, commonly rejected by modern amplifiers (Martín-Vázquez et al., 2013). To avoid this limitation, our analyses of SEEG-ICs rely on relative amplitudes (Fig. 3*a*), either one condition different from zero or differences between conditions (“old” and “new”). This relative amplitude is unaffected by ICA.

### Concluding remarks

The brain circuit of memory encoding and retrieval is an open question in neuroscience (Thompson and Kim, 1996; Ferguson et al., 2019). Our results provide new insights about the role of the hippocampus in recognition memory, linking the hippocampal activity to early stages of recognition memory (∼290 ms). However, it remains unknown which is the specific contribution of the hippocampus to these fast processes. Future investigations will explore the role of the identified hippocampal sources in different memory processes, as well as the specific substructures contributing to the recorded activity. In addition, we have proved the efficacy of ICA to disentangle neuronal generators in SEEG. This opens new possibilities, not only in the analysis of the human hippocampus, but for all intracerebral studies. The use of ICA in other brain structures may reveal the dynamics of different but spatially overlapped structures, overcoming the limitations of traditional montages (Herreras et al., 2015, 2022). Importantly, it can be used in clinical applications with, for example, the potential to identify and separate the generators involved in epileptic networks (Malinowska et al., 2014; Barborica et al., 2021). Further research is granted.

## Synthesis

Reviewing Editor: Anne Keitel, University of Dundee Decisions are customarily a result of the Reviewing Editor and the peer reviewers coming together and discussing their recommendations until a consensus is reached. When revisions are invited, a fact-based synthesis statement explaining their decision and outlining what is needed to prepare a revision will be listed below. The following reviewer(s) agreed to reveal their identity: NONE. Note: If this manuscript was transferred from JNeurosci and a decision was made to accept the manuscript without peer review, a brief statement to this effect will instead be what is listed below.

The reviewers and editor thought that most points were successfully addressed in the revised manuscript, but had a number of remaining concerns and questions. These refer to 1) the interpretation of results, 2) the linking of the manuscript to the wider literature, and 3) some clarifications.

Please see below for unabridged comments by both reviewers, these should be addressed in a point-by-point reply.

*****

Reviewer 1 - Advances the Field (Required)

Conceptual understanding of recognition memory, and hippocampal contributions to recognition memory, does not seem substantially advanced by the work.

Reviewer 1 - Comments to the Authors (Required)

The clarity of the manuscript is largely improved and substantial details have been added. However, I have remaining concerns about the primary interpretations of the work and its relation to the wider literature. The key contribution of this manuscript seems to be the identification of an early hippocampal recognition-related component; however, other work has found early hippocampal contributions to recognition memory and it is not clear that the component reported in the current manuscript reflects the same process and may instead reflect processes unrelated to recognition memory or memory more broadly.

1. Understanding of HC250-IC still seems rather limited and the interpretations made in the manuscript seem too strong given the reported results. The authors find an early dissociation around 290 ms in the other, later component (HC600-IC) and state that this demonstrates "that the hippocampus has an earlier involvement in recognition memory than previously reported." However, there exist reports of early hippocampal responses, e.g. Staresina et al. (2012) in which the hippocampus shows a source effect around 250 ms. Since the current study did not measure recollection/familiarity or source memory, it is not possible to discern whether the early component measured here might similarly reflect source related processing. Given that the dissociation is observed for the later component, it seems possible that HC600-IC is reflecting elements of the LPC. Relatedly, although some work shows slowed reaction times for recollection compared to familiarity judgments, other work (e.g. Gimbel & Brewer (2010), Wixted & Stretch (2004)) has shown the opposite, so it is unclear whether recollection-related brain responses are necessarily slow.

Further complicating the interpretation of the HC250-IC is both the lack of a significant dissociation between old and new responses and the predictability analysis. According to Figure 3A, HC250-IC does not differ between hits and CRs, calling into question whether this reflects recognition-related processing. Furthermore, the authors perform an analysis "to check whether the SEEG-ICs presented a significant activation unrelated to the task predictability," in which they include study-phase data when evaluating HC250-IC. The authors find significant engagement of HC250-IC in this analysis; however, if HC250-IC is recruited during the study phase, then this would strongly suggest that this component does not reflect recognition-related processing. It may not even reflect memory-related processing and may instead be related to visual processing. The hippocampus is thought to be involved in visual perception (e.g. Turk-Browne (2019)) and early hippocampal voltage deflections can be observed in existing recognition data (e.g. Mormann et al. (2005)), potentially reflecting such processing.

2. Several claims are made without appropriate support and in some instances are directly contradicted by the literature.

2a. The authors state in the abstract that, "The hippocampus is considered as a structure with a relatively late involvement in recognition memory, with responses even slower than the behavioral reaction time." Although the hippocampus does show late dissociations, these dissociations still routinely occur prior to the response, e.g. Merkow et al. (2015) which shows hippocampal dissociations around 500-800ms and reaction times > 1000 ms.

2b. The claim in the introduction that "the first difference between old and new elements" in hippocampus occurs "almost 500 ms after stimulus onset" does not appear to be based on a direct test comparing late to early time intervals, but seems based on a hippocampus vs. perirhinal comparison. A lack of significance in earlier time intervals does not mean that a response is specific to later intervals. Indeed, other work has shown hippocampal source recognition effects around 250 ms (Staresina et al. (2012) referenced above), indicating that hippocampus can be involved early relative to stimulus onset.

2c. The authors state "the bipolar montage would measure a combination of the activities of both structures. This overlap can have critical consequences. ... Although this approach has so far been restricted to animal studies;" however, human studies have utilized a bipolar montage, including the Merkow paper cited above.

3. There are still some issues of clarity regarding vague language and analyses/interpretations that appear to be based on visual inspection.

Page 15: "Four different types of independent components (SEEG-ICs) were identified in most of the electrodes" Instead of "most" the authors should report the N/N electrodes in which the components were identified.

Page 19 "The visual inspection suggested that their temporal dynamics were different, with an earlier activation time associated with the familiar items. Therefore, we further explored the modulation of this component."

Page 19 "No correlation between the raster plot and the RT could be visually appreciated"

*****

Reviewer 2 - Advances the Field (Required)

The authors have addressed all my concerns and done the control analysis showing that the two components had significant responses even use the trials involving the predictability to less extent. The results are therefore solid to disentangle both temporal and spatial dynamics of the hippocampal activity in human recognition memory.

Reviewer 2 - Comments to the Authors (Required)

I thank the authors addressed all my concerns. I have some minor comments that I hope the authors could further clarify:

1. When DG (dentate gyrus) was first time mentioned in the Introduction (p.3 ln.69), it should be spelled out.

2. p.9 ln. 214: what did the artifactual single points refer to? Did they mean the significant data points but not survived after LFDR?

3. p.10 ln.245 - 246: why to include the trials from the encoding phase? If including trials from the encoding phase, how could the correct responses be compared between old and new items since there were no new items in the encoding phase and no memory task for the encoding phase?

4. p.12 ln.303 - 304: how did the surrogated ERP was selected? Was it circular shifted or use the 2 s ERP from the starting point randomly selected, which means it could include the data from the next trial?

5. p.15 ln. 355: what were the artefacts? IED or other artefacts? This needs to be clarified in the Methods section.

6. p.18 ln. 444 - 460: were the statistical tests done as same as in the ICA approach?

7. p.18 ln 451: '...we did not identify any significant P250 at the group level' - what is P250? Did the authors mean Hc250-IC or any other ERP component?

8. p.19 ln 461 - 464: was this difference between old and new conditions around 400 - 500 ms of Hc600-IC based on the smaller sample (14 trials) or all trials?

9. p.19 Analysis of hippocampal dynamics: this was only done to Hc600-IC, was it because the Hc250-IC was not significant at group level? I think better explicitly state here or in the Methods.

10. p.25 ln. 615: 'As stated before, it is unlikely that the Hc250-IC potential can be detected from the scalp'. This statement needs to be supported by relevant references.
