# Supplementary figures and images for: Identification of Early Hippocampal Dynamics during Recognition Memory with Independent Component Analysis
Source: eNeuro. 2024 Mar 29;11(4):ENEURO.0183-23.2023. doi: 10.1523/ENEURO.0183-23.2023 (PMC10993203; doi:10.1523/ENEURO.0183-23.2023)

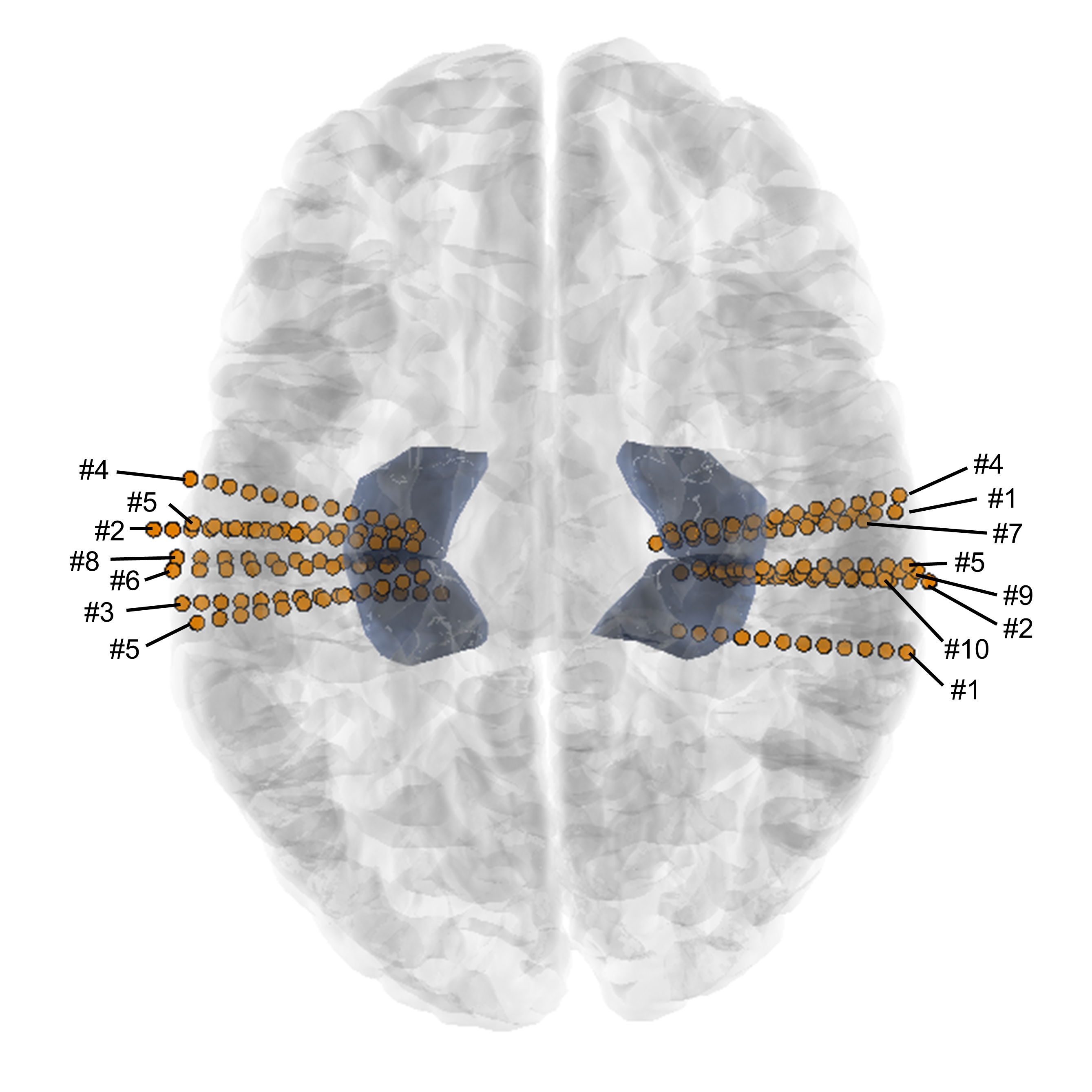

Supplement: Figure 1-1 — Location of all the electrodes Orange dots represent the channel location for each electrode in MNI space. The number indicates the patient. The shaded area depicts the hippocampus. Download Figure 1-1, TIF file. [file eneuro-11-ENEURO.0183-23.2023-s003.tif]

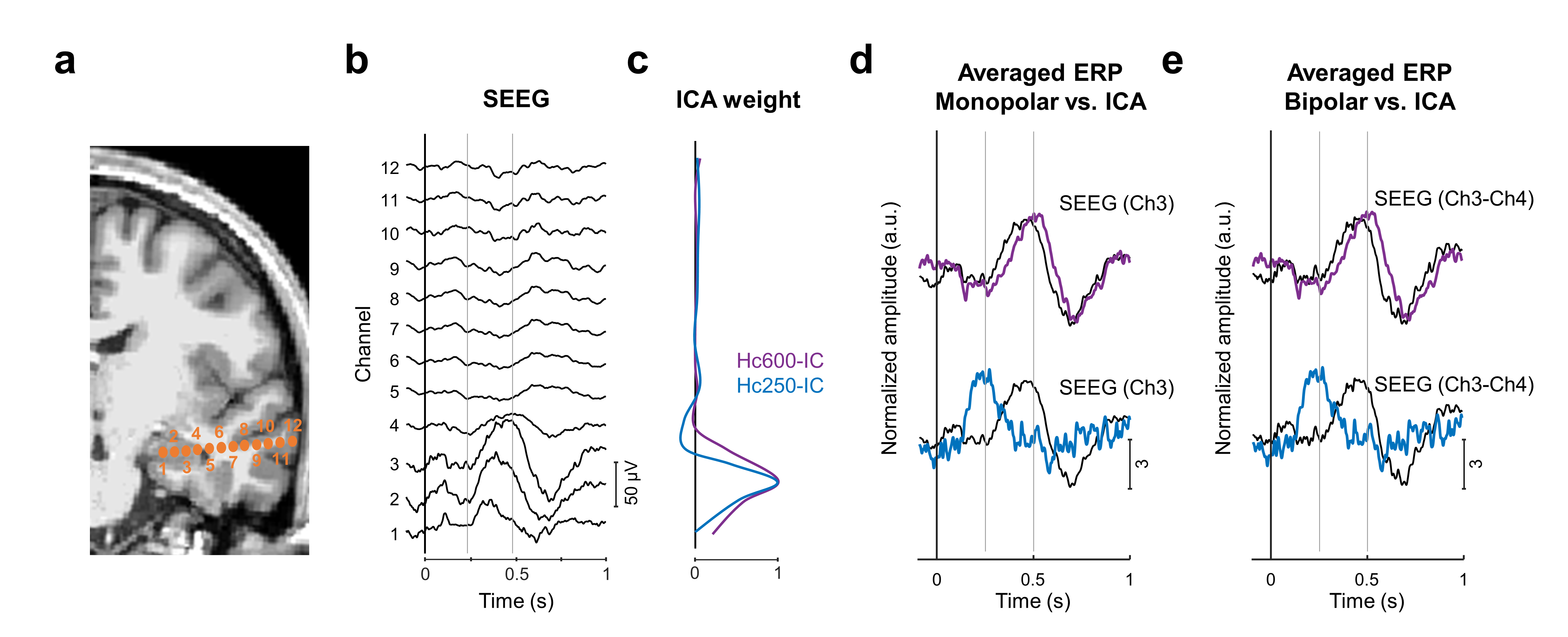

Supplement: Figure 1-2 — Separation of colocalized hippocampal sources in SEEG with ICA a) MRI (3D T1) with reconstruction of SEEG electrode for patient 3. The location of each recording site is represented with orange points.b) Averaged ERP for old responses at each recording site (referential montage).c) Spatial profile of the SEEG-ICs across the electrode. Both components are maximal at the same contact, but their spatial profiles differ.d) Averaged ERP for old responses of SEEG-ICs (color-coded traces) superimposed with the referential montage at the location of maximal contribution from each SEEG-IC (channel 3, black traces. While Hc600-IC correlates with the SEEG response, the early response from Hc250-IC cannot be appreciated in the raw SEEG.e) Same as panel d, but with a bipolar montage for SEEG. As both sources are colocalized close to channel 3, the local currents obtained with the bipolar montage represent the main current generator (Hc600-IC), which hides the activity from Hc250-IC. Note that ICA was always computed on the referential montage. Download Figure 1-2, TIF file. [file eneuro-11-ENEURO.0183-23.2023-s004.tif]

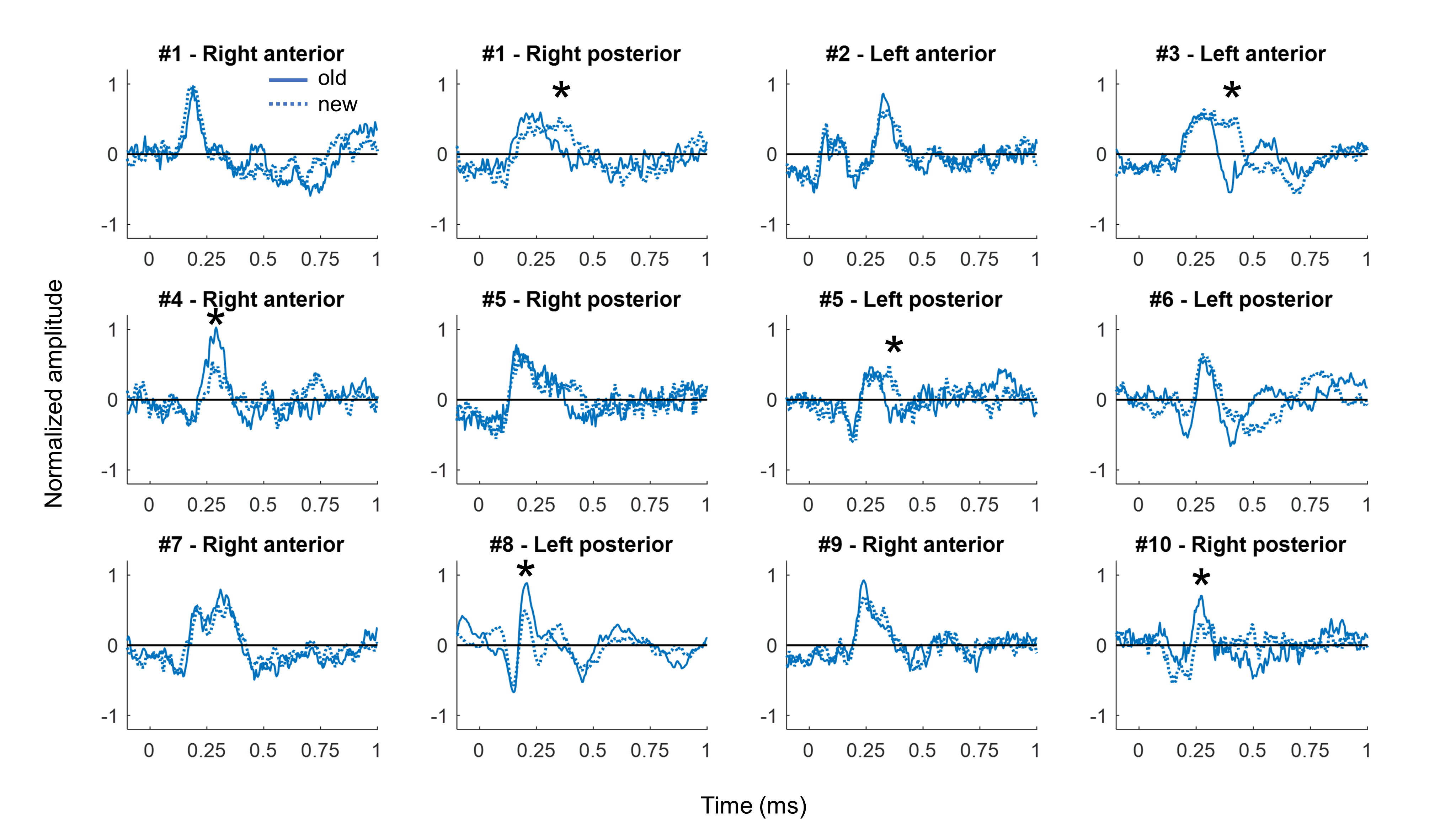

Supplement: Figure 3-1 — Single-case comparison between old and new responses of Hc250-IC Each plot represents the averaged ERP of Hc250-IC during old (solid traces) and new (dashed traces) trials for each electrode. Hashes represent patient number and stars indicate significant differences in amplitude between conditions (* p < 0.05, t-test across trials corrected with LFDR). Only 6 out of 12 electrodes presented a modulation to the memory protocol. In three cases, this difference was due to higher amplitudes after the presentation of old images at early latencies (electrodes 5, 10 and 12). In the other three electrodes, the responses were different, with the responses to the new images standing high during a longer period (electrodes 2, 4 and 7). Download Figure 3-1, TIF file. [file eneuro-11-ENEURO.0183-23.2023-s005.tif]

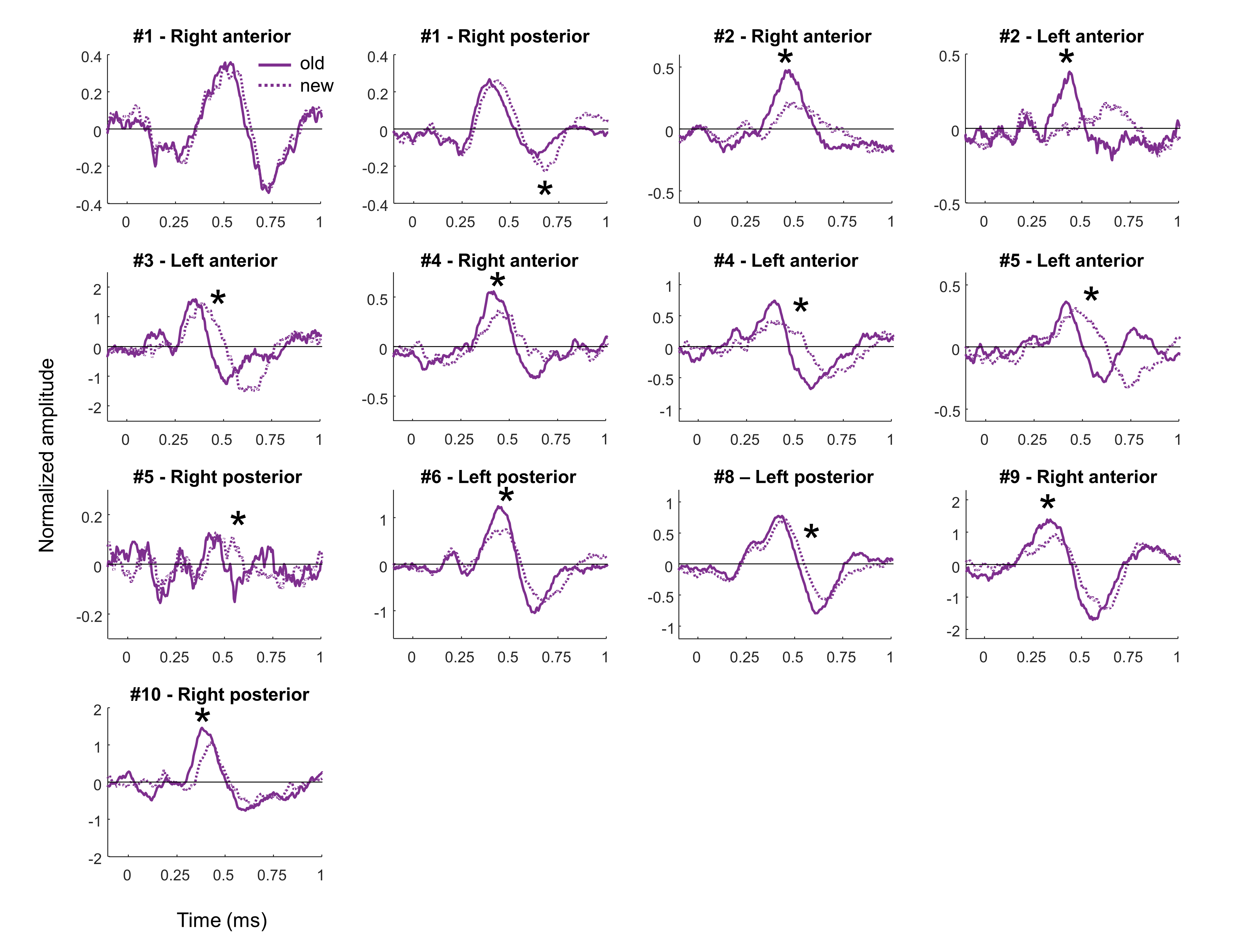

Supplement: Figure 3-2 — Single-case comparison between old and new responses of Hc600-IC Each plot represents the averaged ERP of Hc600-IC during old (solid traces) and new (dashed traces) trials for each electrode. Hashes represent patient number and stars indicate significant differences in amplitude between conditions (* p < 0.05, t-test across trials corrected with LFDR). A total of 12 out of 13 electrodes presented a modulation to the memory protocol. Download Figure 3-2, TIF file. [file eneuro-11-ENEURO.0183-23.2023-s002.tif]
